# Analyzing histopathological images using fused CNN features based on the geometric active contour method for early diagnosis of lung and colon cancer

**DOI:** 10.1007/s12672-025-03907-z

**Published:** 2025-11-05

**Authors:** Yousef Asiri, Ebrahim Mohammed Senan, Hanan T. Halawani, Ibrahim Abunadi, Aisha M. Mashraqi, Eman A. Alshari

**Affiliations:** 1 Department of Computer Science, College of Computer Science and Information Systems, Najran University, Najran, 61441 Saudi Arabia; 2 Department of Artificial Intelligence, Faculty of Computer Science and Information Technology, Al-Razi University, Sana’a, Yemen; 3 Department of Computer Science, College of Applied Sciences, Hajjah University, Hajjah, Yemen; 4 Department of Software Engineering, College of Computer and Information Sciences, King Saud University, P.O. Box 103786, Riyadh, 11543 Saudi Arabia; 5 Computer Science and Information Technology Department, Thamar University, Dhamar, Yemen

**Keywords:** CNN, RF, DT, Lung and colon cancer, ACO, GAC, Fusion features

## Abstract

Lung and colon (LC) cancers are among the most deadly types of cancer, often resulting in death. However, the chances of survival significantly increase with the early detection of these cancers. This study addresses the challenge of early detection of LC tumors, which is crucial for effective treatment. LC cancer is one of the most common tumors that require early detection. However, these tumors appear similar in their early stages, making it challenging for doctors to differentiate between them. To overcome this challenge, we propose an AI-based diagnostic model for detecting LC tumors. Artificial intelligence (AI) techniques have been employed to address this challenge. This study proposes two histopathological image analysis strategies for the early detection of LC cancer. The first strategy involves the diagnosis of LC cancer using decision tree (DT) and random forest (RF) networks with CNN model features, namely ResNet50, DenseNet169, and MobileNet, based on geometric active contour (GAC) and ant colony optimization (ACO) algorithms. The second strategy involves the diagnosis of LC cancer using DT and RF classifiers with the features of combined CNN, namely ResNet50-DenseNet169, DenseNet169-MobileNet, DenseNet169-MobileNet, and ResNet50-DenseNet169-MobileNet, based on the GAC and ACO algorithms. All strategies have demonstrated good results for early stage LC cancer detection. The RF network, which utilized the combined features extracted from the ResNet50- MobileNet-DenseNet169 models, demonstrated notable performance with an AUC of 99.7%, sensitivity of 99.72%, accuracy of 99.8%, precision of 99.76%, and specificity of 99.78%.

## Introduction

LC cancer is the most prevalent and deadly malignancy worldwide. Both cancers are characterized by complex interactions among genetic, environmental, and lifestyle factors [1]. Lung cancer occurs when abnormal cells in the lungs grow uncontrollably and develop into tumors. The most common type of lung cancer is Lung Squamous Cell Carcinoma (SCC), Lung Benign Tissue and Lung Adenocarcinoma [2]. Smoking is the predominant etiological factor for lung carcinoma, constituting approximately 80% of all incidences [3]. Lung cancer pathogenesis disrupts normal cellular processes owing to mutations in critical genes. These mutations drive uncontrolled cell growth, immune surveillance evasion, and angiogenesis, leading to tumor development [4]. In 2020, there were approximately 2.21 million instances of lung cancer, with an estimated 1.8 million deaths from lung cancer worldwide [5]. Colon cancer arises from the accumulation of abnormal genetic mutations in the cells of the colon’s mucosa. Numerous elements, such as genetic factors, play a role in the onset of this disease. Genes play a major role in determining an individual’s susceptibility to colon cancer. Genetic syndromes, such as Lynch syndrome, significantly increase the risk of colon cancer. An increase in red meat consumption and obesity is associated with an increased risk of colon cancer. Gene mutations lead to tumor growth in the colon [6]. In 2020, 1.9 million people were diagnosed with colon cancer, of whom 916,000 died [5]. The incidence of LC cancer at the same time is attributed to several factors, including smoking, family history, and exposure to carcinogens [7]. These factors increase the risk of developing these two types of LC [8]. Several methods are available for diagnosing LC. Biopsies and tissue analyses are important methods for determining the type and grade of cancer. A sample of the cancer is taken for analysis under a microscope, allowing doctors to see abnormal cells and determine the grade of cancer. Biopsy analysis is important for determining the degree of cancer and for selecting the appropriate treatment, whether surgical intervention, radiotherapy, or chemotherapy [9]. Manual diagnosis of pathological tissues has many limitations, such as the scarcity of experienced doctors and differences in doctors’ opinions. Therefore, AI technologies can help address these limitations [10]. CNN models have superior capabilities for extracting hidden and subtle features from LC dataset images. Tissue images contain enormous amounts of information that are difficult to interpret manually, making CNN models important for analysis [11]. CNN models extract highly representative features of cancerous and non-cancerous types that are difficult to analyze manually. Therefore, CNN models contribute significantly to accurate diagnosis. CNN models process large amounts of images with high speed and accuracy. This speed is important in healthcare settings because timely diagnosis saves lives. Differences in interpretation and manual diagnosis may lead to death. Therefore, CNN models have a unified approach to image analysis and diagnosis. In this study, images will be improved, cells of interest will be extracted, separated from healthy cells, and fed to multiple CNN models. Features are derived from the CNN and combined to form highly representative features. The ACO algorithm is applied for high feature reduction and only task selection and classification using ML algorithms.

The significant contributions of this study can be summarized as follows:

- Enhancing histopathological images within the LC25000 dataset by applying fusing filters: the average and Laplacian filters.
- The GAC algorithm was applied to discern infected histopathological tissues, effectively segregating them from healthy tissues and archiving them in the LC25000-ROI dataset.
- Fusion of features from many CNN models, dimension reduction using ACO, and classification using DT and RF networks.
- A hybrid model that synergistically combines the strengths of CNN-DT and CNN-RF systems based on GAC and ACO algorithms was applied.

The remainder of this paper is organized as follows: Sect. 2 reviews relevant previous works, including their methods and findings. Section 3 outlines the strategies and materials used for the histopathological image analysis of LC. Section 4 displays the performance of the strategies and highlights the most significant results. Section 5 discusses the strategies and compares them with relevant studies. Finally, Sect. 6 concludes the study.

## Related work

In this section, we provide a comprehensive review of previous studies that have contributed to the development of methods for the detection of LC. These studies employed various methodologies, tools, and data analysis techniques to elucidate the complexities of LC.

Al-Mamun et al. [12] presented a Cyclic learning rate (CLR) that boosts accuracy and maintains efficiency in the LC25000 dataset. Different pre-trained transfer learning methods with the CNN-from-scratch model. The system increased the total accuracy to 97%. Kumar et al. al. [13] compare handcrafted features (HF) and feature extraction (DFE) for LC cancer classification. The RF classifier with CNN-extracted features achieved the best results, with an accuracy and recall of 98.60%. Wahid et al. [14] presented a study that used pre-trained CNNs: GoogLeNet, ResNet18, and custom CNN. ResNet18 achieved 98.82% accuracy for lung cancer. The customized CNN achieved an accuracy (88.26%) colon cancer and 93.02% for lung cancer, with a longer training time than the other two models. Chehade et al. [15] introduced a system for 5 colon/lung tissue types via histopathological image analysis. Machine learning models offer interpretability owing to feature engineering, whereas Deep Learning (DL) is less interpretable. The ML models showed precise results. XGBoost achieved 98% accuracy. Raju et al. [16] presented a strategy to create a diagnostic system for colon and lung cancers using DL. A hybrid framework was developed: the PCA method was used to reduce the extracted features, and rider optimization with the extreme learning machine (ELM) method was used to train the ELM model. Mengash et al. [17] presented an MPADL-LC3, a technique using marine predator’s (MP) + DL for LC cancer classification: CLAHE pre-processing, MobileNet for feature vector, MP hyperparameter optimization. Simulations on benchmarks showed improved outcomes. The results showed that the MPADL-LC3 technique achieved a precision of 98.18% and a recall of 98.17%. Attallah et al. [18] models for early cancer detection using multiple lightweight DL models. The framework includes feature reduction using PCA and the discrete wavelet transform (DWT). Models: ShuffleNet, MobileNet, and SqueezeNet. DL features combined using DWT fusion and PCA concatenation. The MobileNet, SqueezeNet, and ShuffleNet architectures achieved classification accuracies of 97.1%, 95.3%, and 93.3%, respectively. Tummala et al. [19] presented EfficientNetV2 models with the principles of gradual learning and compound. The GradCAM technique was used to create visual-saliency maps. These maps show the regions in the image that are significant for prediction. Hadiyoso et al. [20] proposed a DL approach using CNN-VGG16 and CLAHE for automatic LC cancer classification. CLAHE boosts detection consistently across epochs. The method achieved a maximum accuracy of 98.96%. Mesut et al. [21] a CNN approach usin Equilibrium, DarkNet-19, SVM and Manta Ray Foraging was applied to classify LC cancer histopathological. The Manta Ray Foraging algorithms were employed to select the most effective features, resulting in % overall accuracy of 96.91%. Sanidhya et al. [22] presented a shallow NN architecture was applied to classify histopathological for the lung, and colon. Satvik et al. [23] a modified pre-trained CNN for LC cancer detection using histopath images. CNN models were trained on the LC25000 dataset. GradCAM and SmoothGrad were used to visualize attention images for malignancy detection. The model achieved an accuracy of 96%. Sameh et al. [24] an enhanced DL for histopatholoy images using a novel fine-tuned network for LC cancer. Regularization, batch normalization, and hyperparameter optimization were performed. The system classifying the LC25000 dataset achieved exceptional metrics: accuracy 97.6% and precision 94.22%. Mumtaz et al. [25] applied a method with histopathology to develop a diagnosis system for LC cancers. The network includes a convolutional layer block with convolutional layers for processing raw images and separable convolutional layers for handling images. Mohammed et al. [26] applied ANN-GoogLeNet for early diagnosis of LC25000 histological images. The enhanced images increased the contrast of the affected area. CNN models generate high-dimensional features. The ANN-GoogLeNet yielded an accuracy of 95.5%.

Sakr et al. presented various CNN-based models for nodule detection and classification from CT scans and histopathological images. AS modified lightweight end-to-end deep learning strategy based on CNNs for lung cancer diagnosis using histopathology images [27]. Patharia et al. utilized a VGG16 CNN model for lung tissue images, demonstrating remarkable precision in discerning cancerous conditions. Another study augmented DenseNet201 with additional layers for lung cancer detection, achieving an average accuracy of 95% [28]. Abhishek et al. presented a hybrid model combining CNN features with other techniques have also shown high accuracy, such as a hybrid ensemble feature extraction approach for colon cancer classification using histopathological images [29]. ViTs are gaining prominence in medical image analysis, particularly for their ability to handle various tasks. While they are data-intensive, fine-tuned ViT models, sometimes integrated with CNNs, have been developed for smaller medical datasets [30]. Katar et al. proposed a hybrid model for Non-Small Cell Lung Cancer classification that combines EfficientNet-B0, LBP, and a ViT encoder [31]. Narasimha et al. utilized a ViT model in combination with CNNs and semantic segmentation methods, such as DeepLabV3 [32]. Wang et al. presented a multi-view CNN model developed to predict the resolution of new intermediate-sized lung nodules in CT screening. senan et al. presented a model that combined three two-dimensional (2D) CNN models and one three-dimensional (3D) CNN model, achieving an Area AUC of 0.81, with a mean sensitivity of 0.63 and a mean specificity of 0.93. This multi-approach significantly improved performance compared to individual 2D, 2.5D, or 3D models [33]. Hasan et al. presented a lightweight and multi-scale CNN model for detecting lung and colon cancers, which is suitable for real-time applications owing to its low parameter count of 1.1 million [34].

Despite these advancements, several challenges and gaps persist in the existing research on lung and colon cancer diagnosis using histopathological images. Many current deep learning models require substantial computational power and resources, and often rely on separate models for feature extraction or diagnosis. The study addresses some of these identified gaps by proposing a novel approach that integrates fused CNN features with the geometric active contour method. Geometric active contour methods are generally employed for accurate object boundary extraction and robustness to noise in images, which is crucial for medical image segmentation. By focusing on fused CNN features, the study aims to overcome limitations in feature extraction and representation that hinder classification accuracy in existing CAD systems. The integration of diverse feature sets from CNN models, potentially enhanced by methods like the geometric active contour, can provide a more comprehensive and accurate representation of subtle patterns in histopathological images. This approach has the potential to contribute to more precise and efficient early diagnosis, ultimately leading to better patient outcomes and treatment planning by assisting medical professionals in quickly and accurately identifying malignancies.

## Materials and methods

### LC25000 dataset description

**Fig. 1:**
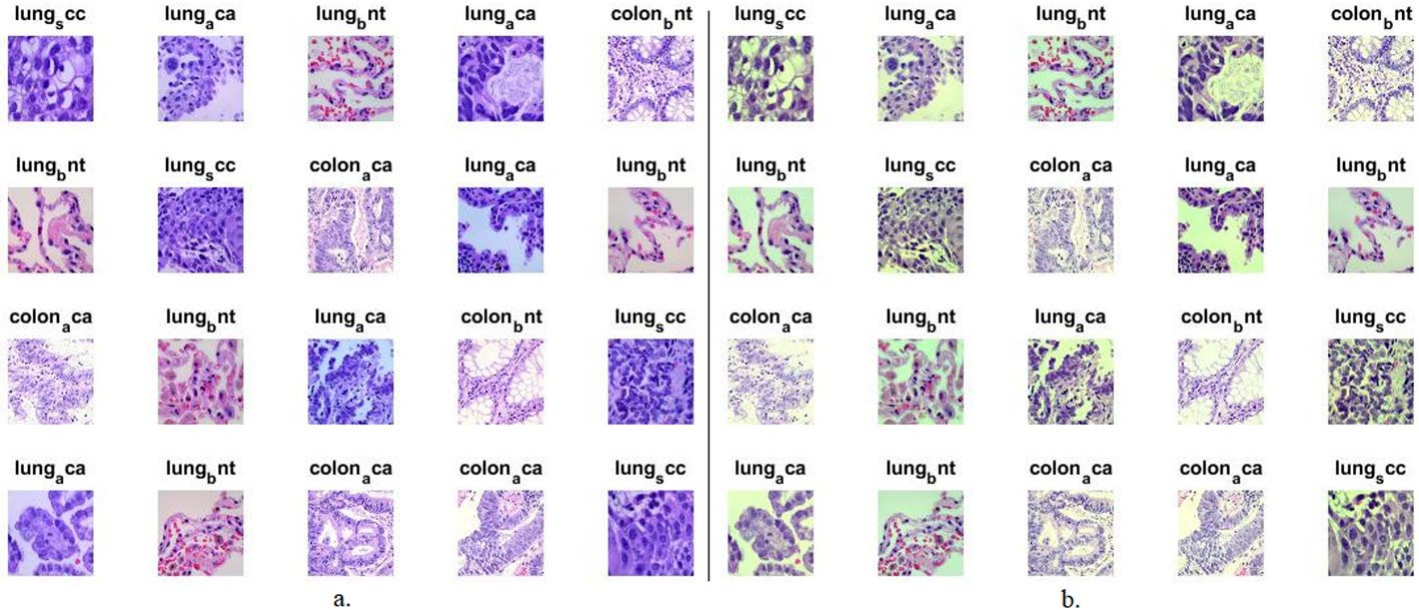
A set of images for the LC25000 data set (a) Before improving histological images (b) After improving histological images.

Within the scope of this study, a dataset comprising histopathological images of instances of LC cancer, denoted as LC25000, was amassed from the publicly accessible Kaggle platform to assess the methodologies. Assembled by Andrew Borkowski and collaborators at James Hospital, Tampa, Florida, this dataset includes 25,000 images. These images were distinctly categorized into colons and three types of lung cancer. The images were uniformly distributed among these five types, thereby establishing a balance within the dataset. Each type contained 5,000 images. The displayed types were designated as colon-aca (representing adenocarcinoma of the colon), colon-bnt (representing Benign Tissue of the colon), lung-aca (representing adenocarcinoma of the lung), lung-bnt (representing Benign Tissue of the lung), and lung-scc (representing SCC of the lung) [35]. The dataset consisted of three distinct classes: those exhibiting malignancy and two classes exhibiting benign characteristics. Notably, Colon Adenocarcinoma accounts for over 95% of all colon cancer occurrences, typically arising from the non-removal of polyps within the large intestine. Similarly, lung adenocarcinoma constitutes more than 40% of lung cancer incidences, manifesting within glandular cells and propagating within the pulmonary structures and alveoli. Lung SCC, contributing to over 30% of cases, is the second most prevalent form of lung cancer, primarily manifesting within the bronchial regions. The remaining two types are benign manifestations that lack the propensity for metastasis. This is visually represented in Fig. 1a displays exemplars from the LC25000 dataset’s five distinct classes.

### Enhancing histological images

Enhancing the quality of histological images, on LC cancers, holds substantial significance due to several crucial reasons: Images are vital for accurate cancer diagnosis. Improving the quality of these images helps AI techniques to identify subtle nuances and cellular patterns that go unnoticed in lower-quality images. High-quality datasets are essential for training accurate algorithms with the increasing use of AI and machine learning in medical diagnostics. Algorithms that detect, segment, and classify cancerous tissues rely on rich, well-annotated datasets. By enhancing histological images, the effectiveness of these algorithms will be improved, leading to more precise and reliable automated diagnostic tools.

In this study, an average filter was used to eliminate artifacts. A Laplacian filter was used to identify regions with low contrast [36]. In the original image, the value of each pixel is substituted with the mean of its surrounding pixels. The filter was configured as a 5 × 5 operator, indicating that it replaced each pixel value with the average of 25 neighboring values, as shown in Eq. 1 [37].

This size was chosen to be sufficiently large to effectively smooth the noise while preserving the larger histological structures critical for diagnosis.

1 $$\:y\left(i\right)=\frac{1}{N}{\sum\:}_{j=0}^{N-1}z\left(j-\text{i}\right)\:\:\:$$

where *y (i)* is input, $\:z\left(j-\text{i}\right)\:$is initial input, and N is total pixel count.

In this study, a Laplacian filter was used to show the pixel intensity changes in the edges and internal structures of an image. A second-order derivative kernel with a positive center ([0, 1, 0; 1, -4, 1; 0, 1, 0]) was used to enhance the edges and fine details in low-contrast tissue regions. This is because the operator is highly sensitive to intensity changes, making the cell boundaries and nuclear details more pronounced. In tissue images, it is difficult to identify edges because there are no differences in intensity; therefore, this filter helps increase the contrast [38]. By emphasizing edges, the Laplacian filter enhances the visibility of fine details that may otherwise be obscured in areas of low contrast. The Laplacian filter provides insights into tissue structural and compositional changes by appearing edges, as shown in Eq. 2.

2 $$\:{\nabla\:\:}^{2}\:f(i,j)=\frac{{\partial\:\:}^{2}\:f}{{\partial\:}^{\:}{i}_{2}}+\:\frac{{\partial\:\:}^{2}\:f}{{\partial\:}^{\:}{j}_{2}}\:\:$$

In the given differential equation of the second order, the symbol $\:\:{\nabla\:\:}^{2}\:f$corresponds to the Laplacian operator, and the indices i and j denote the spatial coordinates of pixels.

Ultimately, the result yielded by the averaging filter is integrated with the result obtained from the Laplacian filter through a subtraction operation involving the respective outcomes of these filters, as demonstrated in Eq. 3.

3 $$\:output\:=y\left(i\right)-\:{\nabla\:\:}^{2}\:f(i,j)\:\:$$

Figure 1b illustrates some of images derived of the LC25000 dataset subsequent to the application of enhancement techniques.

### Geometric active contour method

Image segmentation involves partitioning an image into distinct regions to extract meaningful data. In medical imaging, such as histological images of LC, segmentation is crucial for identifying and isolating regions of interest, such as infected tissues. The GAC algorithm uses geometric features to guide the contours toward regions of interest. Initial points are placed around the regions of interest to form an initial curve [39]. The curve is continuously updated to move toward the cancerous tissue (ROI) based on the internal and external forces, as shown in Eq. 4. The internal forces smoothen the internal curve. External forces affect the curves and jagged edges. Forces capture tissue properties, such as color, texture, density, edges, and changes in texture [40].

4 $$\:{G}_{cv}C=\underset{outside}{\int\:}\left(I\right(x)-{m}_{\:1}{)}^{2}\:dx+\:\underset{inside}{\int\:}\left(I\right(x)-{m}_{\:2}{)}^{2}\:dx+\:\beta\:\:length\:(C)\:$$

where *I* mean gray intensity, the designations outside and inside pertain to regions exterior and interior to the delineating contour *C*, while m1 and m2 specifically denote the mean intensities of the external and internal domains, respectively. The symbol β corresponds to a configurable parameter, and length (*C*) signifies the extent of contour *C*. Notably, *G*cv embodies a discerning propensity for initialization alongside a subset of requisite parameters for configuration. The function of operator G is to orchestrate the movement of the contour towards the confines of the epithelial tissue boundary of LC cancer.

The GAC algorithm was used to segment infected tissues from histological images of LC cancer using the following steps:

- The initial curve was placed around the infected tissue.
- The image gradient force was calculated. The image gradient force measures the intensity of changes in an image. The gradient is high at the edges of the objects; therefore, the image gradient force attempts to move the curve towards the edges of the infected tissue.
- The curvature force was calculated. The curvature force is a measure of the smoothness of a curve. The curvature is high at sharp turns; therefore, the curvature force attempts to keep the curve smooth.
- The curve is updated to move towards the object boundaries based on the image gradient and curvature forces.
- Steps 2–4 were repeated until the curve converged to the object boundaries.

**Fig. 2:**
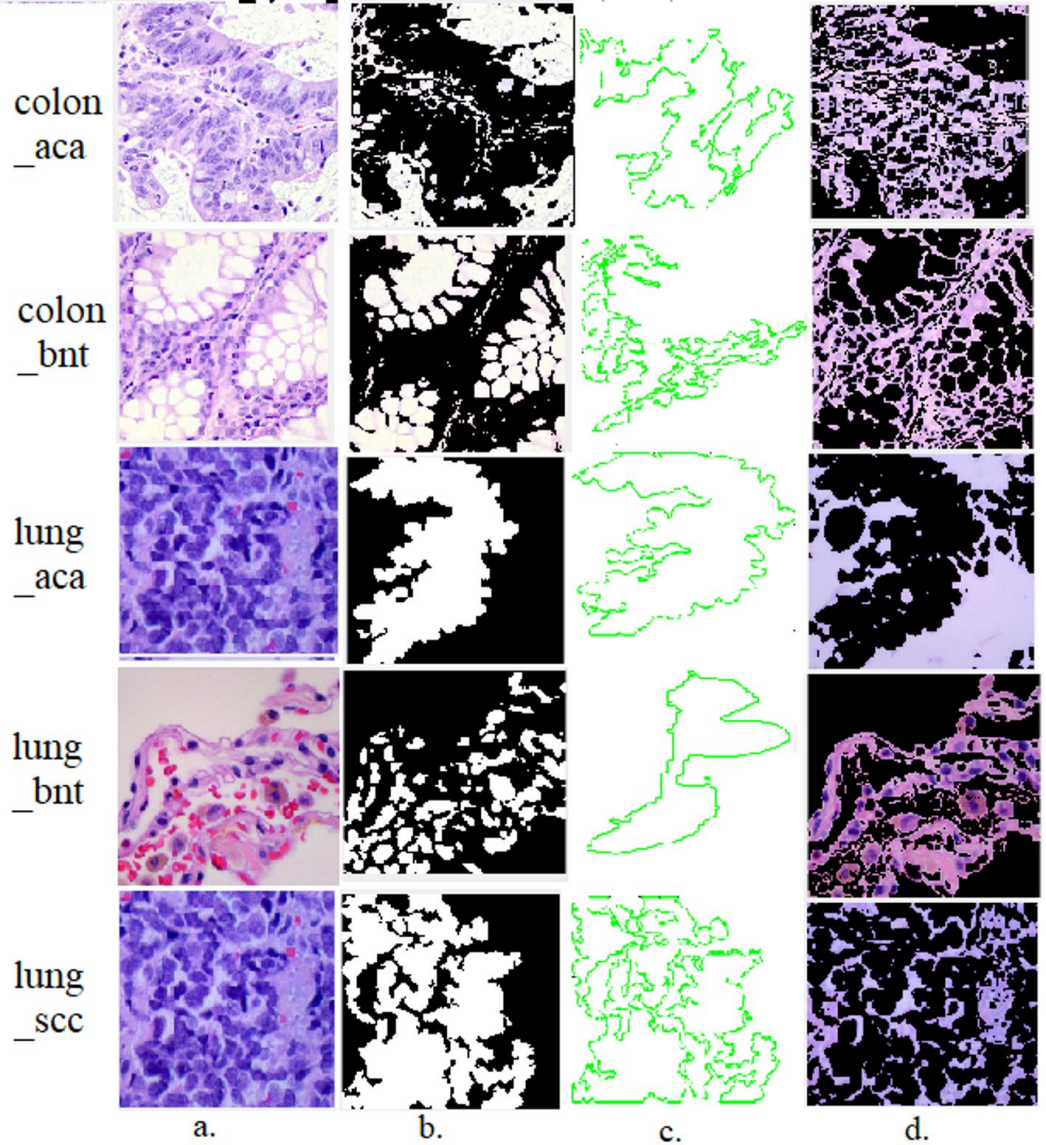
A sample of the classes of the LC25000 dataset after histological segmentation. **a** Original images **b** Segmentation of histological images. **c** Selection of infected histological tissues. **d** Region of interest

The GAC algorithm is a promising tool for segmenting infected tissues in histological images. The GAC algorithm was used to segment infected tissues from LC cancer histological images with good results. Finally, regions of interest in histological images of LC were saved within new folders and subsequently input into CNN models to extract intricate attributes from tissue-afflicted epithelia. Figure 2 illustrates the efficacy evaluation of the GAC algorithm in delineating regions characterized by diseased epithelial tissue, effectively demarcating them from healthy tissue.

### Extract CNN feature

Extracting deep and complex features from histological images of LC cancer involves utilizing DL techniques, particularly CNNs. These networks are well-suited for extracting hierarchical features directly from images for image analysis tasks. The process of using convolutional layers to extract these features and their application to histological images is described below [41]. They detect various features, such as textures, shapes, and edges, from the input images. Convolutional layers are composed of adjustable filters, also known as kernels, that move across the input image to execute convolutions. The convolution process entails element-wise effect integration of the filter with a segment of the input image, followed by summation of the resultant values [42]. This process allows the model to learn the local patterns and features present in the image. In this work, Feature Extraction from Histological Images: In histological images of LC cancer, the goal is to automatically identify and extract features indicative of cancerous tissues or other important characteristics. These features may include the arrangement of cells, cell nuclei, tissue structures, and more subtle aspects that human experts may not easily detect [43]. CNNs excel in hierarchical feature learning. The initial layers identify basic features, such as corners and edges, whereas the more advanced layers capture intricate and abstract elements, such as textures and shapes. This progression allows the network to gradually build a representation of an image from basic to complex features. Throughout the training phase, the CNN modifies the weights of its filters to reduce the discrepancies between its predictions and true labels. This fine-tuned the filters to recognize cancer-related features. Images are typically represented as matrices when convolutional layers are applied. Each image channel corresponds to a specific staining or color channel used in histology [44]. Convolutional filters are designed to slide over these channels, capturing features across the spatial dimensions of the image. As the filters slide, they perform convolutions at every position, capturing local patterns, as shown in Eq. 5 [45]. The outcome yielded by the convolutional stratum comprises an array of feature maps, where each map accentuates the detection of distinct attributes in the input image. Deeper layers in the network focus on more complex features by combining information from multiple filters in the previous layers [46].

5 $$\:y\left(t\right)=\left(x\text{*}f\right)\left(t\right)=\int\:x\left(a\right)f\left(t-a\right)\:\:da\:\:$$

In this context, *f (t)* denotes the filter, *x (t)* the input image, and *y (t)* the resulting output.

The reduction of feature maps generated from convolutional layers is often performed using pooling layers in CNNs. Pooling layers diminish the features while preserving the salient information of utmost significance [47].

Max-pooling is a pooling technique that operates on individual patches of the feature map. The feature maps were divided into rectangular regions, and the maximum value was chosen from each rectangle. A max-pooling operation entails moving a small window across a feature map. At each window position, the maximum value within the window was selected and placed in the pooled output [48]. The window moves by a certain stride (e.g., 2) to the next position, resulting in a reduced-size feature map, as shown in Eq. 6.

Similar to max-pooling and average-pooling operations, which entail moving a window across the feature map [49], at each window position, the average values within the window are computed and placed in the pooled output. Similar to max-pooling, the window moves by a certain stride to produce a reduced-size feature map, as shown in Eq. 7. Average pooling provides a form of downsampling that maintains a broader input representation [50].

6 $$\:z\left(i;\:j\right)={max}_{m,n=1\dots\:.k}\:f\left[\left(i-1\right)p+m;\:\left(\:j-1\right)p+n\right]$$

7 $$\:z\left(i;\:j\right)=\frac{1}{{k}^{2}}\sum\:_{m,n=1\dots\:.k}f\left[\left(i-1\right)p+m;\:\left(\:j-1\right)p+n\right]\:$$

where the *f* is the filter within the spatial image domain, the parameter *k* represents the count of pixels, the indices *m* and *n* denote the specific matrix coordinates and the variable *p* corresponds to the magnitude of the p-step increment.

In this study, ResNet50, DenseNet169, and MobileNet were used to extract high-efficiency feature for histological images of LC cancer. The ResNet50, DenseNet169, and MobileNet models generate higher-level feature with sizes of (3, 3, 512), (32, 32, 640), and (7, 7, 1024). These features are then converted into feature vectors using the Global Average Pooling (GAP) layer, reducing the feature dimensionality. GAP layer is used in CNNs to transform high-level feature into a single, low-level feature vector. It’s typically applied at the end of a CNN architecture before the fully connected layers, often replacing the traditional flattening and fully connected layers. The main purpose of the GAP layer is to generate a compact and informative representation of the entire input feature map. The input to the GAP layer is high-level feature generated by the previous convolutional layers. These features contain spatial information about various features detected in the input image. The GAP layer outputs feature vectors are 1024, 1664, and 2048 for the ResNet50, DenseNet169, and MobileNet models, respectively. Finally, the feature vectors of all images in the LC25000 dataset are concatenated into a feature matrix with sizes of 25,000 × 1024, 25,000 × 1664, and 25,000 × 2048, respectively.

The features of CNN models are multidimensional feature vectors that capture critical patterns in lung and colon cancer images. These features include spatial hierarchies, texture information, and object-level details that play a fundamental role in distinguishing between healthy and cancerous tissues. These vectors are numerical representations (floating-point numbers) that capture the complex visual patterns in the image. The range of these features typically lies between 0 and 1, as the values are often normalized after activation functions, such as ReLU, are applied. However, in some layers, the range may vary, especially before normalization. All features were represented as floating-point numbers (float32), which are suitable for neural network processing and ensure precision during the optimization and training phases. The features undergo normalization; therefore, the values generally lie within the range [0, 1].

### Ant colony optimization algorithm

CNN models often produce high-dimensional feature sets. In this study, the CNN models extracted features from high-level LC cancer histological images. Therefore, one of the feature reduction techniques must be applied. In this study, the ACO algorithm was applied to reduce the high-level dimensions. ACO is a nature-inspired optimization algorithm inspired by the foraging behavior of ants. It is commonly used to solve combinatorial optimization problems, including feature selection and dimensionality reduction [51]. The ACO reduces the high features of CNN and represents them in low-dimensional feature vectors by selecting the essential features. Important features are those that are most relevant to the classification task. The ACO algorithm was used to remove unimportant and redundant features that did not contribute to the classification accuracy. Ants are attracted to other ants that have a high level of pheromones. The pheromone trails are constantly talking, helping other ants search for the best subset of features [52]. The ACO algorithm has proven to be highly efficient in reducing CNN features, resulting in high classification accuracy.

ACO reduces the features of CNN.

- Step 1: The ants are initialized using a random set of features.
- Step 2: The pheromone trails guide the ants in their search for the best feature subsets. The pheromone trails are initially set to a low value and are updated as the ants explore the feature space.
- Step 3: The ants move through the feature space by following pheromone trails. Each ant chooses the feature with the highest pheromone level at each step.
- Step 4: The pheromone trails are updated after each ant moves. The pheromone level of a feature is increased if the ant selects that feature and is decreased if the ant does not select that feature.
- Step 5 involves the iteration of Steps 2 to 4 until the fulfilment of a termination condition, which reaches a threshold for the error rate or attains a specified maximum count of iterations.

When constructing a solution, each ant considers both the quality of the features (indicated by their pheromone levels) and the importance of the features. Features with higher pheromone levels are more likely to be selected. This reflects the positive feedback nature of ant behavior: ants are more likely to follow the paths that other ants have taken before. Each ant erects a solution by selecting features based on pheromone levels and the importance of the features. Once each ant erects a solution, the performance of the solution is evaluated using a predefined objective function.

The goal of ACO in this study was to identify and retain the features most strongly associated with early signs while eliminating those that were weakly correlated or irrelevant to lung and colon cancers. In the ACO algorithm, each ant navigates the feature space and selects features based on their importance for detecting cancer. Features with a higher probability of representing significant cancer-related characteristics are reinforced via pheromone trails, meaning they are more likely to be selected by other ants. Features that are less relevant or redundant are less likely to be selected, thereby improving the overall feature subset used for classification.

### Build training templates

Medical image processing is marked by the classification phase, which is the final stage of assigning categorical labels to processed images. The attainment of optimal classification accuracy in this pivotal stage is intricately intertwined with the effectiveness and proficiency exhibited by the preceding processing stages [53]. Following image optimization and the extraction of pathological histology from the LC25000 dataset through a segmentation algorithm, the resulting pathological histological samples underwent comprehensive analysis via DL models to derive high-dimensional features. Subsequently, the dimensions of these feature maps were progressively reduced using an iterative process facilitated by the ACO algorithm, which judiciously selected and pruned salient features. These features were then channeled into DT and RF networks for classification.

The selection of tree-based algorithms, specifically DT and RF, was guided by several key advantages they offer in the context of this study’s pipeline:

The RF algorithm quantitatively ranks the importance of features. This is crucial for this study, as it allows us to identify which specific features extracted from the CNNs (e.g., textures, nuclear patterns, and glandular structures) are the most discriminative for classifying different cancer types. This provides valuable and clinically relevant insights beyond mere classification accuracy.

By using CNNs as fixed feature extractors and passing the optimized features to DT/RF models, we leveraged the representational power of deep learning while utilizing the extreme computational efficiency of tree-based algorithms for the final classification. This leads to significantly faster training and inference times for the final models.

Because RF creates many decision trees on random subsets of the data with random subsets of the features (and “bagging” and “feature randomness”), it has less variance and generalizes better than a single DT. This feature of robustness is critical to ensure that the model’s performance is consistent and not related to quirks in the training set, which is superior for hypothetical clinical use.

#### Decision tree algorithm

The DT classifier is vital for classifying the features of CNN models extracted from LC cancer stromal images. This method works by dividing the datasets into smaller sub-datasets. This is achieved by constructing a tree, where the inner nodes represent the features and the leaves represent the final class [51]. This method enables the learning of features and relationships within the feature space, thus classifying the features [54]. The algorithm continues to partition the data until each subset is represented by a single class. In classifying the features of CNN for images of LC cancer, the DT method receives the features of ResNet50, DenseNet169, and MobileNet. It also receives the hybrid features of the ResNet50-DenseNet169, DenseNet169-MobileNet, DenseNet169-MobileNet, and ResNet50-DenseNet169-MobileNet models. The dataset is classified into its class.

#### Random forest algorithm

The RF algorithm is a powerful method for the feature classification of CNN models of histological images of LC cancer. This algorithm works on the basis of ensemble learning by creating many DT. In this study, the algorithm generated several DT, each whichree trained a random subset of the data. When all trees are created, they collectively contribute to the classification [55]. Each tree votes for the expected class, and the final classification is selected based on the majority votes. The significance of the RF algorithm is manifold in classifying the LC cancer histological images. By aggregating the predictions of multiple DT, the algorithm provides a more robust and accurate classification of histopathological features. The diversity among trees helps capture different aspects of complex cancer-related patterns in the data. RFs inherently measure the importance of each feature by assessing the extent to which they contribute to the ensemble’s predictive power [56]. This information is valuable for understanding the relevance of the different features in cancer classification. The RF algorithm receives the features of the ResNet50, DenseNet169, and MobileNet models. It also receives the fused features of the ResNet50-DenseNet169, DenseNet169-MobileNet, and DenseNet169-MobileNet models [57].

### Implementation strategy of proposed systems

**Fig. 3:**
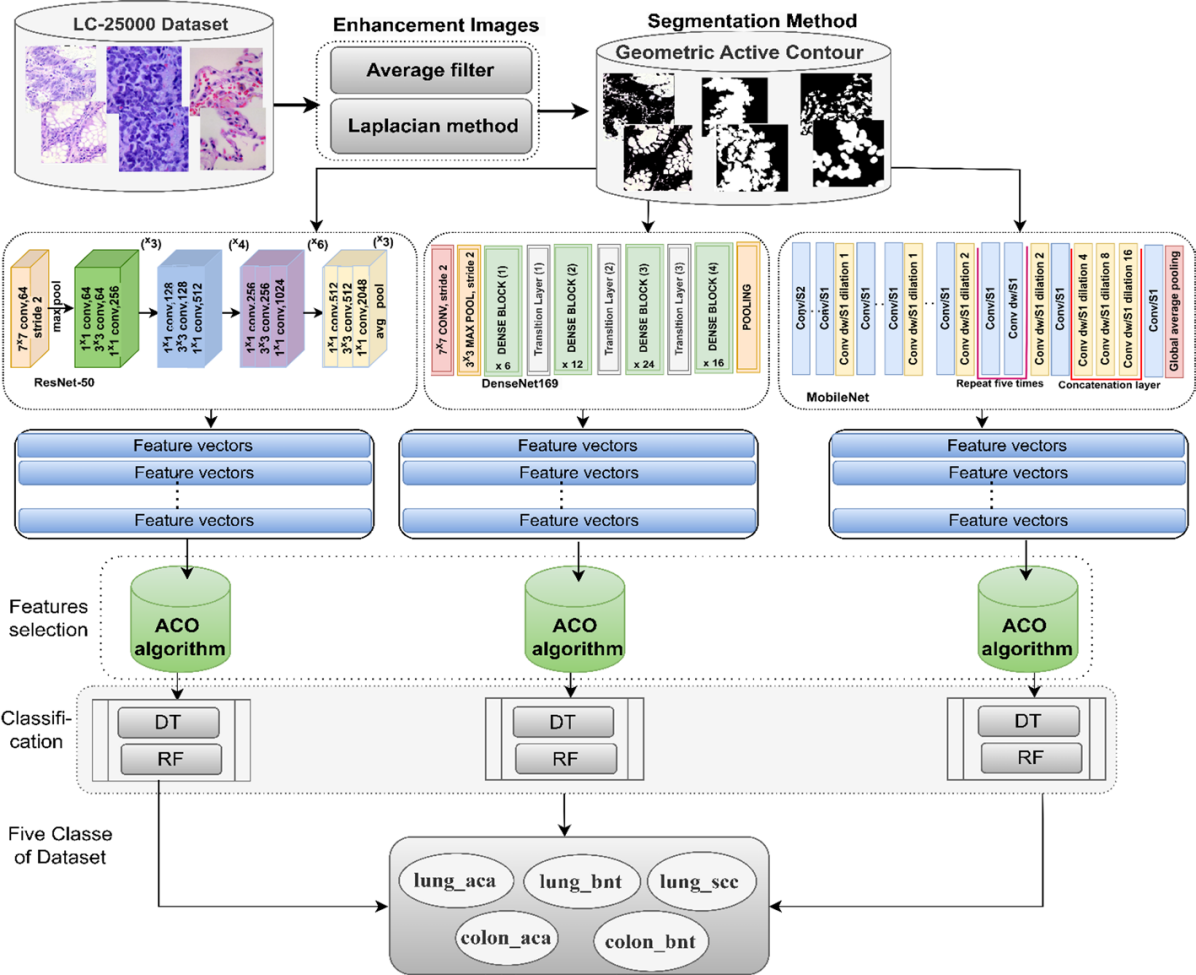
Diagnosis of histological images of the LC25000 data set of LC cancer by a hybrid method between CNN with DT and RF networks

The proposed methodology for classifying images of the LC25000-ROI dataset for LC cancer involves utilizing a hybrid model that combines machine learning techniques with CNN models based on the GAC algorithm and feature selection ACO algorithm. The working framework, as shown in Fig. 3, encompasses the following steps.

Histological Image Enhancement: Commencing with a set of LC25000 data, the images underwent an optimization process to enhance the visibility of low-contrast pathological histology and mitigate the influence of artifacts. Segmentation and Isolation of Infected Histology: The histological images demonstrating signs of infection were subsequently segregated from their healthy counterparts by applying the GAC segmentation method. These segmented images were then saved in folders known as LC25000-ROI. CNN Model Application: The histological images were individually inputted into the ResNet50, DenseNet169, and MobileNet CNN models [58]. These models extract pertinent features using convolutional layers and pooling operations. Consequently, feature matrices were generated, comprising 25,000 × 1024, 25,000 × 1664, and 25,000 × 2048 for the ResNet50, DenseNet169, and MobileNet models, respectively. Feature Selection by ACO: The features, which possess elevated dimensions, are inputted to the ACO algorithm. This algorithm selects features related to the afflicted tissue while deleting unnecessary and duplicated features. Model Training and Evaluation: The important features are input into the DT and RF networks [59]. These networks are trained and subsequently evaluated to measure their performance in histological image classification.

**Fig. 4:**
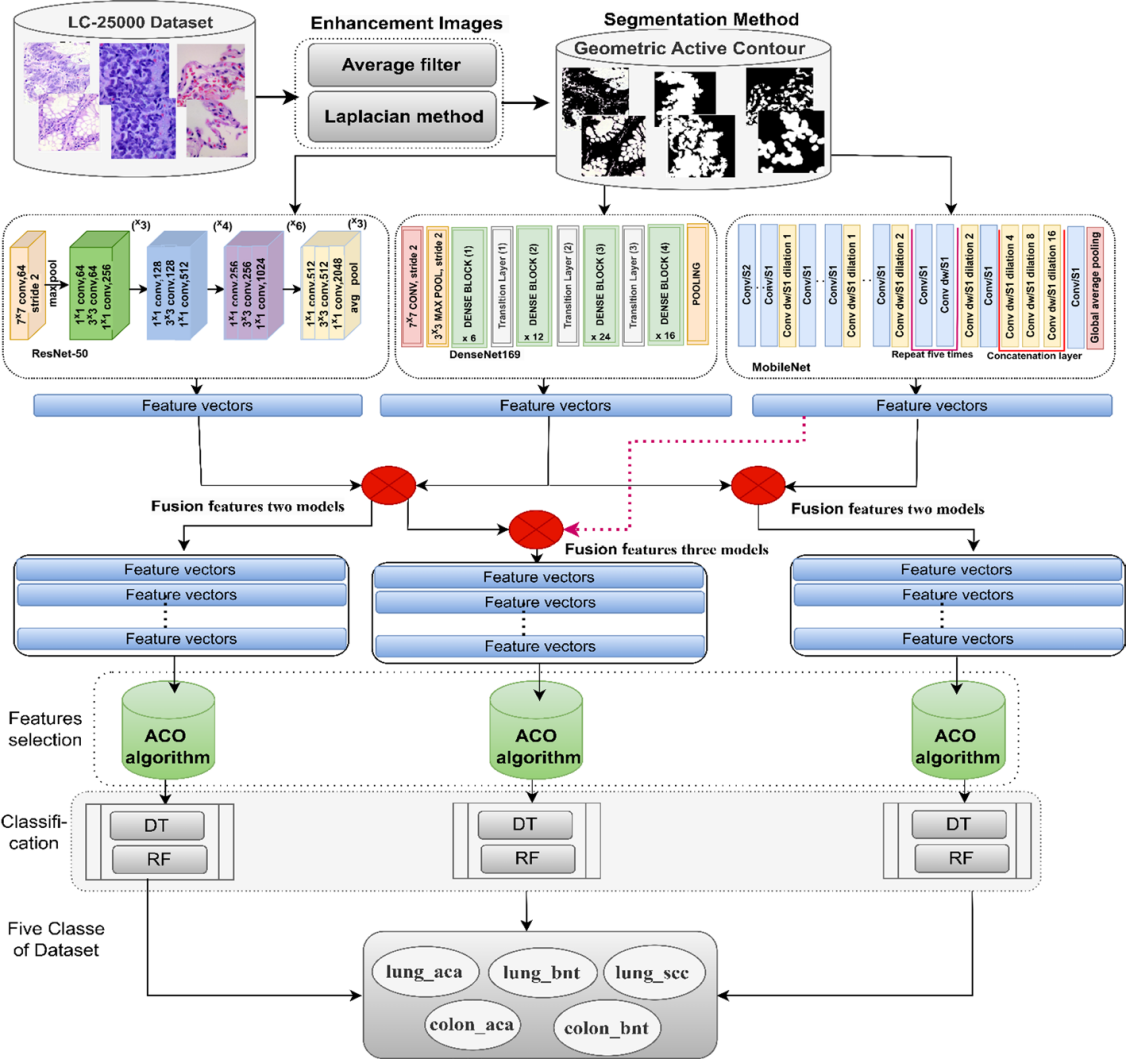
Diagnosis of images of the LC25000 data set of LC cancer by DT and RF networks with fusion features for CNN models

The proposed histological image classification for the LC25000-ROI dataset of LC cancer using a hybrid model of machine learning techniques and fused features extracted from CNN models. The hybrid model is based on the GAC and feature selection ACO algorithms, and the execution through the ensuing phases is illustrated in Fig. 4.

The complete workflow is as follows.

Preprocessing and GAC Segmentation: Input histological images from the LC25000 dataset first underwent contrast enhancement. The GAC model was then applied to precisely segment and isolate the ROIs containing pathological tissue from the image background. These segmented ROIs were saved and constituted the refined LC25000-ROI dataset used for all subsequent analyses.

CNN-based Feature Extraction: Each image from the LC25000-ROI dataset was processed independently using three pretrained CNNs: ResNet50, DenseNet169, and MobileNet. We used the activations from the final pooling layers of each network as a high-level feature vector representing the image. This results in three distinct feature matrices.

ResNet50: 25,000 samples x 1024 features, DenseNet169: 25,000 samples x 1664 features, and MobileNet: 25,000 samples x 2048 features.

Feature Fusion: The fusion of features from different CNNs was performed through concatenation. The feature vectors extracted from each network for a single image were combined end-to-end into a single comprehensive feature vector.

Feature Selection using ACO: The concatenated feature vectors are high-dimensional. To mitigate the curse of dimensionality and remove redundant information, we employed the ACO algorithm as a feature selection method. ACO identifies and selects the most discriminative subset of features from each concatenated matrix. The final selected feature counts provided in the manuscript (625, 690, 720, and 810 features for the respective fusion combinations) were the results of this optimization process.

Classification: The optimized feature sets obtained after the ACO were used to train and evaluate two distinct classifiers: DT and RF. The performance of these classifiers was then evaluated on the test set.

## Experimental results

### Dataset partitioning and system specifications

In this study, the methods were applied to the LC25000 dataset to detect and distinguish the first stages of LC malignancies. The LC25000 dataset contains 25,000 histological images acquired through biopsy procedures from afflicted tissue sites in patients. This dataset was partitioned into five categories, demarcating malignant and benign tumor instances of LC. The distribution is as follows: 5000 histological renditions of Colon Adenocarcinoma, 5000 histological of Colon Benign Tissue, another 5000 portraying Lung Adenocarcinoma, an additional 5000 histological Lung Benign Tissue, and finally, 5000 histological illustrations representative of Lung SCC. The dataset is characterized by three variants of malignant tumors and two variants of benign tumors. Notably, the dataset demonstrated equilibrium across all classes, each with an identical number of histological images. These techniques involved allocating 60% of the available data for training and validation (90:10), while the remaining 40% was reserved for assessing the algorithm’s performance, as delineated in Table 1.

**Table 1:** Splitting the LC data set in all phases

Phase	60% (90:10)		Testing 40%
Types	Training (90%)	validation (10%)	
Colon Adenocarcinoma	2700	300	2000
Colon Benign Tissue	2700	300	2000
Lung Adenocarcinoma	2700	300	2000
Lung Benign Tissue	2700	300	2000
Lung SCC	2700	300	2000

During the training phase of the lung and colon datasets, we employed a data augmentation strategy to artificially expand and modify the datasets. Data augmentation is a group of methods that create new, slightly modified versions of the original images to enrich the diversity, balance, and robustness of the training dataset, all without collecting additional images, which can be expensive and time-consuming. Data augmentation not only increases the dataset size but also improves the model’s ability to generalize by exposing it to a wider array of variations that could naturally be present in any given original dataset.

For this work, each image in the original dataset was augmented to create five copies of each image for the dataset, yielding a total dataset of 16,200 images per class. The augmentation transformations we used included transformations that reflected real changes that might be observed during the preparation of histopathological slides, such as small rotations, shifts, stretching, and scaling, while maintaining the key pathological features (such as nuclear morphology and glandular structures) intact and diagnostic.

In addition to simple replication, augmentation provides enhancement of the images by applying controlled variability to the image with a number of processes, including:

- Geometric transformations: random rotations, flips (horizontal/vertical), translations, scaling, and elastic deformations to mimic small distortions from tissue sectioning or imaging.
- Photometric transformations: alterations in brightness, contrast, saturation, hue, and color jitter to mimic differences in staining or illumination.
- Cropping and zooming: random or centered crops and magnification that allow the model to see both global and fine-grained tissue structures.
- Blurring or sharpening: differences in the focus of the slide or calibration of the microscope can be mimicked to allow the algorithm to tolerate variability in imaging conditions.

In the end, these processes of augmentation yield a more robust representation of possible input variations, so that the model overfits less, generalizes better, and the classifier learns semantically meaningful configurations, as opposed to engaging in rote memorization of artifacts specific to the datasets.

### Systems performance measures

The confusion matrix is important for evaluating the classification systems used in the LC25000 dataset. A confusion matrix is a tabular representation that compares the expected (true) classes with the predicted (actual) classes of a dataset, thereby enabling the calculation of performance metrics. The entries in this matrix indicate the number of cases that were classified accurately and imprecisely. The main diagonal of the matrix represents accurately classified cases, called true positives (TP), while the remaining cells include Incorrectly classified cases, which include false negatives (FN) and true negatives (TN). The effectiveness of these systems is measured using Eqs. 9–14.

9 $$\:\text{AUC}\:=\frac{\text{TP}\:\text{Rate}}{\text{FP}\:\text{Rate}}$$

10 $$\:\text{S}\text{e}\text{n}\text{s}\text{i}\text{t}\text{i}\text{v}\text{i}\text{t}\text{y}=\frac{\text{T}\text{P}}{\text{T}\text{P}+\text{F}\text{N}}\:\text{*}100\text{\%}$$

11 $$\:\text{A}\text{c}\text{c}\text{u}\text{r}\text{a}\text{c}\text{y}=\frac{\text{T}\text{N}+\text{T}\text{P}}{\text{T}\text{N}+\text{T}\text{P}+\text{F}\text{N}+\text{F}\text{P}}\:\text{*}100\text{\%}\:$$

12 $$\:\text{P}\text{r}\text{e}\text{c}\text{i}\text{s}\text{i}\text{o}\text{n}=\frac{\text{T}\text{P}}{\text{T}\text{P}+\text{F}\text{P}}\:\text{*}100\text{\%}\:$$

13 $$\:\text{S}\text{p}\text{e}\text{c}\text{i}\text{f}\text{i}\text{c}\text{i}\text{t}\text{y}=\frac{\text{T}\text{N}}{\text{T}\text{N}+\text{F}\text{P}}\:\text{*}100\text{\%}\:$$

14 $$\:F1-score\:=\frac{2\times\:(Precision\times\:Sensitivity)}{Precision+Sensitivity}$$

### Results of pre-trained CNN

This section introduces the performance outcomes of the pretrained CNN, including ResNet50, DenseNet169, and MobileNet. These models were trained on the extensive ImageNet dataset, encompassing more than 1,200,000 images designed to categorize over 1,000 distinct classes. However, the ImageNet dataset lacks representation from numerous biomedical image datasets, such as those containing histological images of LC cancer. These models leverage the knowledge accumulated during training on the ImageNet dataset to perform novel tasks involving the classification of histological images of LC cancer. The input layers accept images sourced from the LC25000 dataset and channel them through the layers, facilitating intricate feature extraction.

**Fig. 5:**
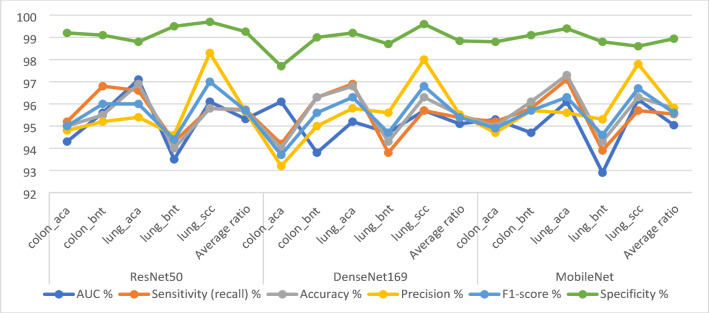
Display the results of CNN for analyzing images of the LC25000 dataset to diagnose and distinguish between LC cancers

Table 2; Fig. 5 illustrate the outcomes derived from using pre-trained ResNet50, DenseNet169, and MobileNet models in the analysis of histological images, specifically targeting the diagnosis of the LC25000 dataset. The ResNet50 model achieved an AUC of 95.32%. Sensitivity, a critical metric indicative of the model’s ability to accurately detect TP, was 95.74%. The attained accuracy level was 95.7%, indicating correctly classified samples. The precision, which indicates the model’s precision in labeling positive instances, was 95.66%. The specificity, underscoring the model’s capability to identify negative cases, was 99.26%. Conversely, the DenseNet169 model yielded an AUC of 95.1, sensitivity of 95.38%, accuracy of 95.5%, precision of 95.52%, and specificity of 98.84%. Finally, the MobileNet model achieved an AUC of 95.04%, sensitivity of 95.54%, accuracy of 95.8%, precision of 95.82%, and specificity of 98.94%.

**Table 2:** Performance results of pre-trained CNN models for analyzing images of the LC25000 dataset to diagnose and distinguish between LC cancers

Models	Type of lesion	AUC %	Sensitivity (recall) %	Accuracy %	Precision %	F1-score %	Specificity %
ResNet50	colon_aca	94.3	95.2	95	94.8	95	99.2
	colon_bnt	95.6	96.8	95.5	95.2	96	99.1
	lung_aca	97.1	96.6	96.9	95.4	96	98.8
	lung_bnt	93.5	94.3	94	94.6	94.4	99.5
	lung_scc	96.1	95.8	95.8	98.3	97	99.7
	Average ratio	95.32	95.74	95.7	95.66	95.7	99.26
DenseNet169	colon_aca	96.1	94.2	94	93.2	93.7	97.7
	colon_bnt	93.8	96.3	96.3	95	95.6	99
	lung_aca	95.2	96.9	96.8	95.8	96.3	99.2
	lung_bnt	94.7	93.8	94.3	95.6	94.7	98.7
	lung_scc	95.7	95.7	96.3	98	96.8	99.6
	Average ratio	95.1	95.38	95.5	95.52	95.4	98.84
MobileNet	colon_aca	95.3	95.2	95	94.7	94.9	98.8
	colon_bnt	94.7	95.8	96.1	95.7	95.7	99.1
	lung_aca	96.1	97.1	97.3	95.6	96.3	99.4
	lung_bnt	92.9	93.9	94.3	95.3	94.6	98.8
	lung_scc	96.2	95.7	96.3	97.8	96.7	98.6
	Average ratio	95.04	95.54	95.8	95.82	95.6	98.94

### Results of hybrid models of machine learning with CNN

This section summarizes the performance of hybrid models that combine CNN (ResNet50, DenseNet169, and MobileNet) with both DT and RF methods to analyze the LC25000 dataset for LC cancers. The hybrid models segment infected histological tissues after optimizing images and extracting feature maps using CNN models. The ACO method was used to retain important features and eliminate redundant ones. The features generated by ACO were then sent to the DT and RF methods. The hybrid models that integrate CNN_DT and CNN-RF configurations applied to the histological image analysis of LC cancers of the LC25000 dataset present deep capabilities in effectively discerning between instances of LC cancers.

**Fig. 6:**
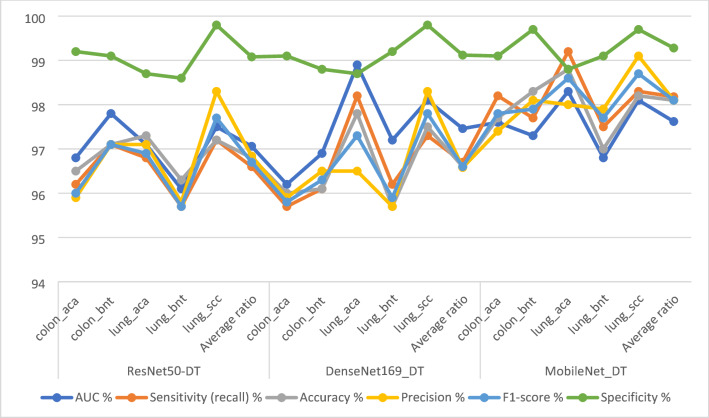
Display of the results of CNN-DT for image analysis of the LC25000 data set for diagnosing and differentiating LC cancer

Table 3; Fig. 6 comprehensively display the evaluation metrics for the CNN-DT hybrid models in the analysis of images of the LC25000 dataset, with the primary objective of early detection of LC cancers. The results are structured as follows: The ResNet50-DT model attained a notable performance with an AUC value of 97.06%, accompanied by a sensitivity rate of 96.6%, an accuracy rate of 96.8%, a precision value of 96.84%, and a specificity rate of 99.08%. Similarly, the DenseNet169-DT model showcases competitive statistics, exhibiting an AUC of 97.46%, a sensitivity of 96.7%, an accuracy rate of 96.6%, a precision rate of 96.58%, and a specificity rate of 99.12%. The MobileNet-DT model yielded distinctive outcomes, with an AUC of 97.62%, sensitivity rate of 98.18%, accuracy of 98.1%, precision of 98.1%, and specificity rate of 99.28%. These results suggest that CNN-DT hybrid models can be effectively used for the early diagnosis of LC cancers. The MobileNet-DT model achieved slightly better performance, followed by the ResNet50-DT and DenseNet169-DT models, respectively.

**Table 3:** Performance results of CNN-DT models for histological image analysis of the LC25000 data set for diagnosing and differentiating LC cancer

Models	Type of lesion	AUC %	Sensitivity (recall) %	Accuracy %	Precision %	F1-score %	Specificity %
ResNet50-DT	colon_aca	96.8	96.2	96.5	95.9	96	99.2
	colon_bnt	97.8	97.1	97.1	97.1	97.1	99.1
	lung_aca	97.1	96.8	97.3	97.1	96.9	98.7
	lung_bnt	96.1	95.7	96.3	95.8	95.7	98.6
	lung_scc	97.5	97.2	97.2	98.3	97.7	99.8
	Average ratio	97.06	96.6	96.8	96.84	96.7	99.08
DenseNet169_DT	colon_aca	96.2	95.7	96	95.9	95.8	99.1
	colon_bnt	96.9	96.1	96.1	96.5	96.3	98.8
	lung_aca	98.9	98.2	97.8	96.5	97.3	98.7
	lung_bnt	97.2	96.2	95.7	95.7	95.9	99.2
	lung_scc	98.1	97.3	97.5	98.3	97.8	99.8
	Average ratio	97.46	96.7	96.6	96.58	96.6	99.12
MobileNet_DT	colon_aca	97.6	98.2	97.7	97.4	97.8	99.1
	colon_bnt	97.3	97.7	98.3	98.1	97.9	99.7
	lung_aca	98.3	99.2	98.8	98	98.6	98.8
	lung_bnt	96.8	97.5	97	97.9	97.7	99.1
	lung_scc	98.1	98.3	98.2	99.1	98.7	99.7
	Average ratio	97.62	98.18	98.1	98.1	98.1	99.28

**Fig. 7:**
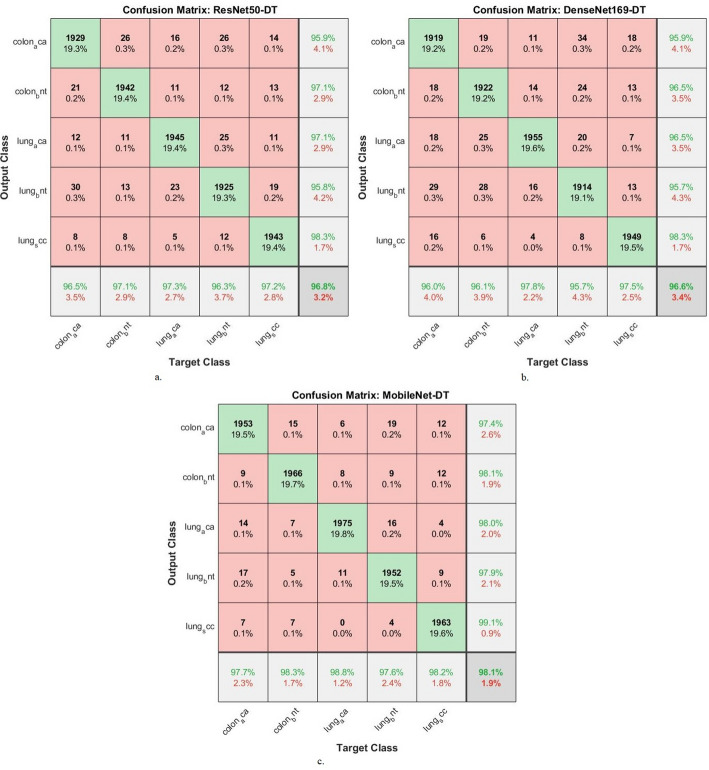
Display of confusion matrix of results of CNN-DT for image analysis of the LC25000 data set for diagnosing and differentiating LC cancer

Confusion matrices were generated using the hybrid models CNN-DT and CNN-RF. Confusion matrices highlighted the efficiency of these hybrid models in the early detection and differentiation of LC cancers. As shown in Fig. 7, the correlation matrix displays the results of the hybrid models. ResNet50-DT achieved an accuracy of 96.5% for colon_aca, 97.1% for colon_bnt, 97.3% for lung_aca, 96.3% for lung_bnt, and 97.2% for lung_scc. DenseNet169_DT achieved an accuracy of 96% for colon_aca, 96.1% for colon_bnt, 97.8% for lung_aca, 95.7% for lung_bnt, and 97.5% for lung_scc. The MobileNet-DT achieved an accuracy of classifying colon_aca of 97.7%, colon_bnt of 98.3%, lung_aca of 98.8%, lung_bnt of 97.6%, and lung_scc of 98.2%.

**Fig. 8:**
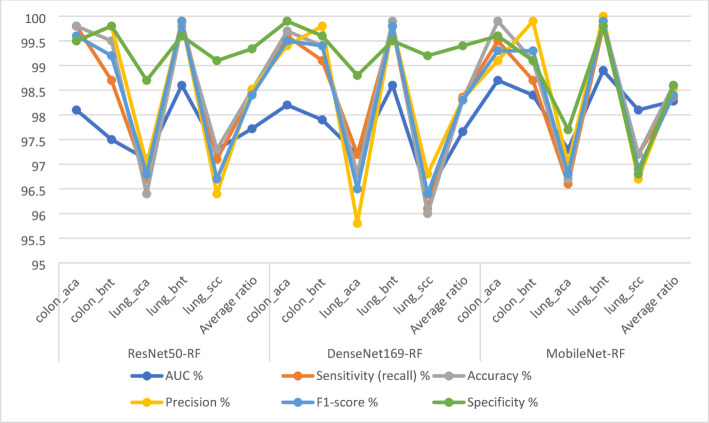
Display of the results of CNN-RF for histological analysis of the LC25000 data set for diagnosing and differentiating LC cancer

Table 4; Fig. 8 show the results of the CNN-RF model for analyzing LC25000 images to detect LC cancers early. esNet50-RF model: AUC (97.72%), sensitivity (98.44%), accuracy (98.5%), precision (98.52%), specificity (99.34%). DenseNet169-RF model: AUC (97.66%), sensitivity (98.36%), accuracy (98.3%), precision (98.32%), specificity (99.4%). MobileNet-RF model: AUC (98.28%), sensitivity (98.36%), accuracy (98.6%), precision (98.54%), specificity (98.6%).

**Table 4:** Performance results of CNN-RF for histological analysis of the LC25000 data set for diagnosing and differentiating LC cancer

Models	Type of lesion	AUC %	Sensitivity (recall) %	Accuracy %	Precision %	F1-score %	Specificity %
ResNet50-RF	colon_aca	98.1	99.8	99.8	99.5	99.6	99.5
	colon_bnt	97.5	98.7	99.5	99.8	99.2	99.8
	lung_aca	97.1	96.7	96.4	97	96.8	98.7
	lung_bnt	98.6	99.9	99.8	99.9	99.9	99.6
	lung_scc	97.3	97.1	97.3	96.4	96.7	99.1
	Average ratio	97.72	98.44	98.5	98.52	98.4	99.34
DenseNet169-RF	colon_aca	98.2	99.6	99.7	99.4	99.5	99.9
	colon_bnt	97.9	99.1	99.4	99.8	99.4	99.6
	lung_aca	97.2	97.2	96.8	95.8	96.5	98.8
	lung_bnt	98.6	99.8	99.9	99.8	99.8	99.5
	lung_scc	96.4	96.1	96	96.8	96.4	99.2
	Average ratio	97.66	98.36	98.3	98.32	98.3	99.4
MobileNet-RF	colon_aca	98.7	99.5	99.9	99.1	99.3	99.6
	colon_bnt	98.4	98.7	99.1	99.9	99.3	99.1
	lung_aca	97.3	96.6	96.7	97	96.8	97.7
	lung_bnt	98.9	99.8	99.9	100	99.9	99.8
	lung_scc	98.1	97.2	97.2	96.7	96.9	96.8
	Average ratio	98.28	98.36	98.6	98.54	98.4	98.6

**Fig. 9:**
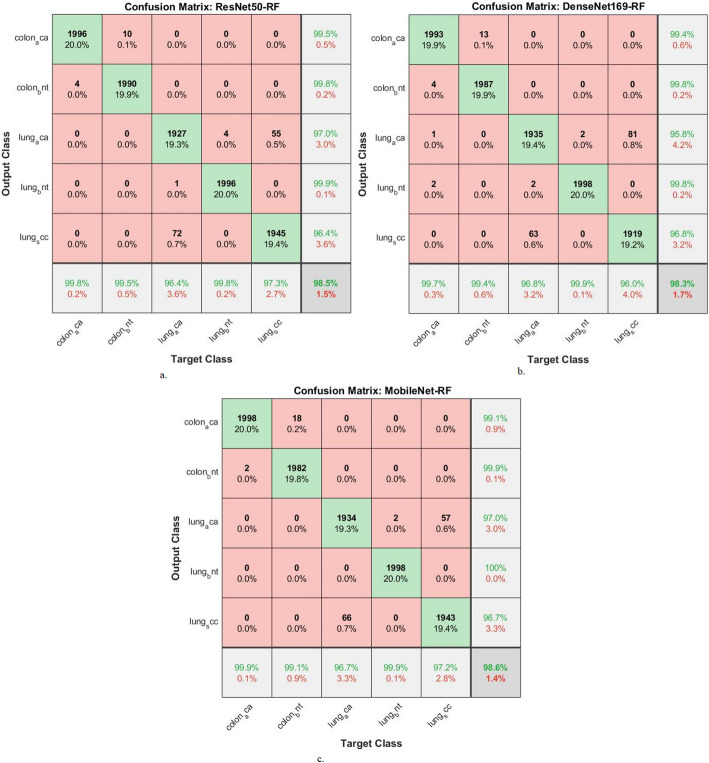
Display of confusion matrix of results of CNN-RF for histological analysis of the LC25000 data set for diagnosing and differentiating LC cancer

Figure 9 shows the correlation matrix that displays the performance of the hybrid models. The ResNet50-RF model achieved accuracies of 99.8% for colon_aca, 99.5% for colon_bnt, 96.4% for lung_aca, 99.8% for lung_bnt, and 97.3% for lung_scc. The DenseNet169-RF model achieved accuracies of 99.7% for colon_aca, 99.4% for colon_bnt, 96.8% for lung_aca, 99.9% for lung_bnt, and 96% for lung_scc. The MobileNet-RF model achieved an accuracy of classifying colon_aca of 99.9%, colon_bnt of 99.1%, lung_aca of 96.7%, lung_bnt of 99.9%, and lung_scc of 97.2%.

### Results of hybrid models of machine learning with fused CNN features

This section presents the results of the DT and RF algorithms with the fused CNN features. These hybridized algorithms were employed to analyze the histological images contained within the LC25000 dataset. The primary focus is on the early diagnosis and distinction between infected tissues for LC cancer types. The images were enhanced, followed by segmentation of the infected pathological tissues using the GAC method. Within this strategy, CNN models assume a pivotal role by ingesting images of infected pathological tissues from the LC25000 dataset. After ingestion, the CNN models engage in the extraction of feature maps via the utilization of convolutional layers and pooling operations. The process of sequentially integrating the features of the CNN models and storing them in the feature array is as follows (ResNet50-DenseNet169, DenseNet169-MobileNet, and ResNet50-MobileNet). The imperative of retaining salient features while eliminating redundant and unimportant ones is achieved by implementing ACO. The outcomes of the ACO method yielded a highly representative feature matrix that strongly correlated with the tissue type affected by each disease. The feature matrix was fed into the DT and RF methods to train and assess the model performance. The hybrid models, predicated upon the amalgamation of the CNN models’ fused features, were tailored to analyze infected pathological tissues within the LC25000 dataset. These models exhibit high proficiency in distinguishing between different classes of infected tissues associated with LC cancer types.

**Fig. 10:**
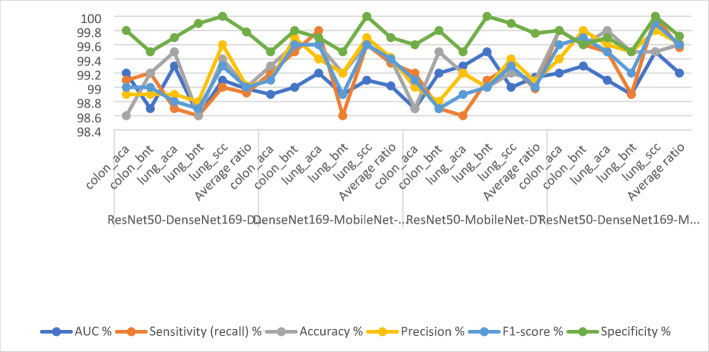
Display of the performance results of DT with fusion CNN features for histological analysis of the LC25000 data set for diagnosing and differentiating LC cancer

Table 5; Fig. 10 show the evaluation of the CNN-DT hybrid models. ResNet50-DenseNet169-DT achieved an accuracy (99%), sensitivity (98.92%), specificity (99.78%), precision (99.02%), AUC (98.98%), 99 The DenseNet169-MobileNet-DT achieved accuracy (99.4%), sensitivity (99.34%), specificity (99.7%), precision (99.42%), and AUC (99.02%). The ResNet50-MobileNet-DT achieved accuracy (99.6%), sensitivity (99.56%), specificity (99.72%), precision (99.62%), and AUC (99.2%). The ResNet50-DenseNet169-MobileNet-DT also achieved an accuracy (99.6%), sensitivity (99.56%), specificity (99.72%), precision (99.62%), and AUC (99.2%).

**Table 5:** Performance of DT with fusion CNN features for histological analysis of the LC25000 data set

Models	Type of lesion	AUC %	Sensitivity (recall) %	Accuracy %	Precision %	F1-score %	Specificity %
ResNet50-DenseNet169-DT	colon_aca	99.2	99.1	98.6	98.9	99	99.8
	colon_bnt	98.7	99.2	99.2	98.9	99	99.5
	lung_aca	99.3	98.7	99.5	98.9	98.8	99.7
	lung_bnt	98.6	98.6	98.6	98.8	98.7	99.9
	lung_scc	99.1	99	99.4	99.6	99.3	100
	Average ratio	98.98	98.92	99	99.02	99	99.78
DenseNet169-MobileNet-DT	colon_aca	98.9	99.2	99.3	99.1	99.1	99.5
	colon_bnt	99	99.5	99.6	99.7	99.6	99.8
	lung_aca	99.2	99.8	99.6	99.4	99.6	99.7
	lung_bnt	98.9	98.6	99.2	99.2	98.9	99.5
	lung_scc	99.1	99.6	99.6	99.7	99.6	100
	Average ratio	99.02	99.34	99.4	99.42	99.4	99.7
ResNet50-MobileNet-DT	colon_aca	98.7	99.2	98.7	99	99.1	99.6
	colon_bnt	99.2	98.7	99.5	98.8	98.7	99.8
	lung_aca	99.3	98.6	99.2	99.2	98.9	99.5
	lung_bnt	99.5	99.1	99	99	99	100
	lung_scc	99	99.3	99.2	99.4	99.3	99.9
	Average ratio	99.14	98.98	99.1	99.08	99	99.76
ResNet50-DenseNet169-MobileNet-DT	colon_aca	99.2	99.8	99.8	99.4	99.6	99.8
	colon_bnt	99.3	99.6	99.6	99.8	99.7	99.6
	lung_aca	99.1	99.5	99.8	99.6	99.5	99.7
	lung_bnt	98.9	98.9	99.5	99.5	99.2	99.5
	lung_scc	99.5	100	99.5	99.8	99.9	100
	Average ratio	99.2	99.56	99.6	99.62	99.6	99.72

The hybrid models integrating CNN-DT and CNN-RF architectures by fused features from the CNN yield confusion matrices that effectively illustrate the result characteristics of these hybrid models in early LC cancer detection and the distinction between them.

**Fig. 11:**
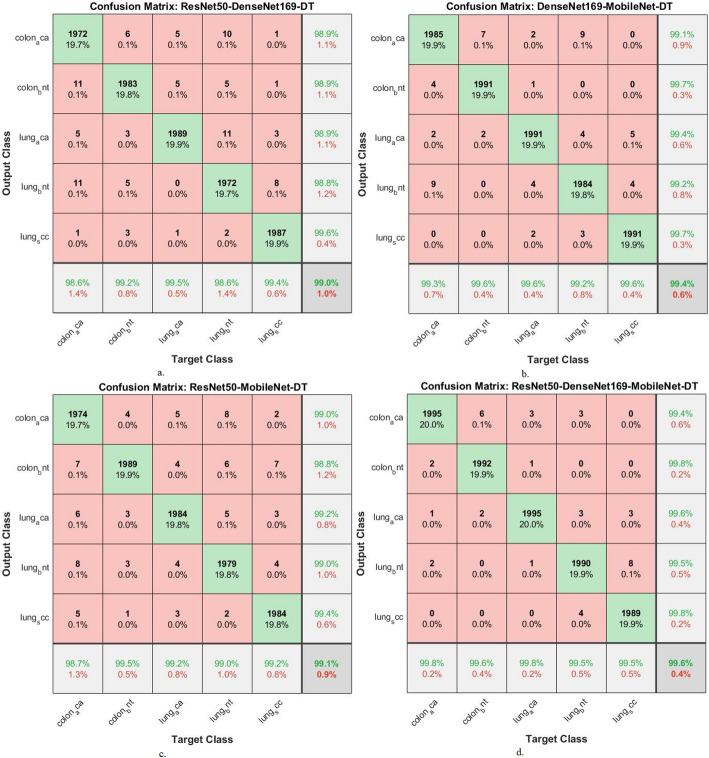
Display of confusion matrix of performance results of DT with CNN features for analysis of the LC25000 data set for diagnosing and differentiating LC cancer

Figure 11 depicts the confusion matrix representing the ResNet50-DenseNet169-DT, DenseNet169-MobileNet-DT, and ResNet50-MobileNet-DT models, all employed for the diagnosis of LC in the LC25000 dataset. This figure illustrates the accuracy metrics for each class. Specifically, the ResNet50-DenseNet169-DT model achieved remarkable accuracy rates for distinct classes: 98.6% for Colon_aca, 99.2% for Colon_bent, 99.5% for Lung_aca, 98.6% for Lung_bnt, and 99.4% for Lung_scc. In contrast, focusing on the classification of fused features extracted from DenseNet169-MobileNet using the DT method, the accuracy achieved for each class was as follows: 98.7% for colon _aca, 99.5% for colon _bent, 99.2% for lung _aca, 99% for lung _bnt, and 99% for lung _scc. Finally, the classification of the fused features extracted from the ResNet50-DenseNet169-MobileNet model using the DT method achieved the following accuracies for each class: 99.8% for colon _aca, 99.6% for colon _bent, 99.8% for lung _aca, 99.5% for lung _bnt, and 99.5% for lung _scc.

**Fig. 12:**
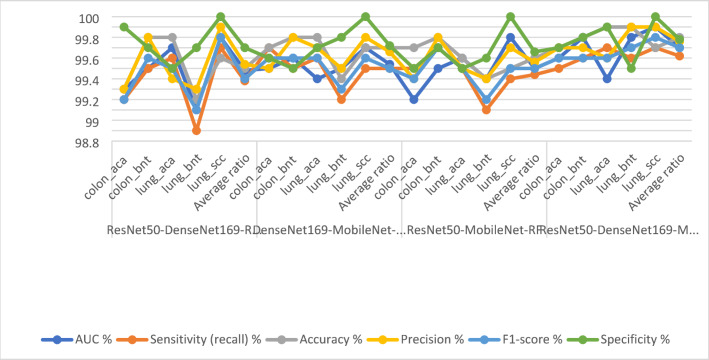
Display of the results of RF with multi- CNN features for histological analysis of the LC25000 data set for diagnosing and differentiating LC cancer

Table 6 with Fig. 12 shows the evaluation of CNN-RF hybrid models. The ResNet50-DenseNet169-RF model achieved accuracy (99.5%), sensitivity (99.38%), specificity (99.78%), precision (99.54%), and AUC (99.48%). The DenseNet169-MobileNet-RF achieved accuracy (99.7%), sensitivity (99.5%), specificity (99.72%), precision (99.6%), and AUC (99.54%). The ResNet50-MobileNet-RF achieved accuracy (99.6%), sensitivity (99.44%), specificity (99.66%), precision (99.56%), and AUC (99.5%). The ResNet50-DenseNet169-MobileNet-RF achieved accuracy (99.8%), sensitivity (99.62%), specificity (99.78%), precision (99.76%), and AUC (99.7%).

**Table 6:** Display of the result of RF with multi-CNN features for histological analysis of the LC25000 data set for diagnosing and differentiating LE cancer.

Models	Type of lesion		AUC %	Sensitivity (recall) %	Accuracy %	Precision %	F1-score %	Specificity %
ResNet50-DenseNet169-RF	colon_aca		99.3	99.2	99.3	99.3	99.2	99.9
	colon_bnt		99.5	99.5	99.8	99.8	99.6	99.7
	lung_aca		99.7	99.6	99.8	99.4	99.5	99.5
	lung_bnt		99.1	98.9	99.2	99.3	99.1	99.7
	lung_scc		99.8	99.7	99.6	99.9	99.8	100
	Average ratio		99.48	99.38	99.5	99.54	99.4	99.7
DenseNet169-MobileNet-RF	colon_aca		99.5	99.7	99.7	99.5	99.6	99.6
	colon_bnt		99.6	99.5	99.8	99.8	99.6	99.5
	lung_aca		99.4	99.6	99.8	99.7	99.6	99.7
	lung_bnt		99.5	99.2	99.4	99.5	99.3	99.8
	lung_scc		99.7	99.5	99.7	99.8	99.6	100
	Average ratio		99.54	99.5	99.7	99.66	99.5	99.72
ResNet50-MobileNet-RF	colon_aca		99.2	99.5	99.7	99.4	99.4	99.5
	colon_bnt		99.5	99.7	99.8	99.8	99.7	99.7
	lung_aca		99.6	99.5	99.6	99.5	99.5	99.5
	lung_bnt		99.4	99.1	99.4	99.4	99.2	99.6
	lung_scc		99.8	99.4	99.5	99.7	99.5	100
	Average ratio		99.5	99.44	99.6	99.56	99.5	99.66
ResNet50-DenseNet169-MobileNet-RF	colon_aca		99.6	99.5	99.7	99.7	99.6	99.7
	colon_bnt		99.8	99.6	99.8	99.7	99.6	99.8
	lung_aca		99.4	99.7	99.9	99.6	99.6	99.9
	lung_bnt		99.8	99.6	99.9	99.9	99.7	99.5
	lung_scc		99.9	99.7	99.7	99.9	99.8	100
	Average ratio		99.7	99.62	99.8	99.76	99.7	99.78

**Fig. 13:**
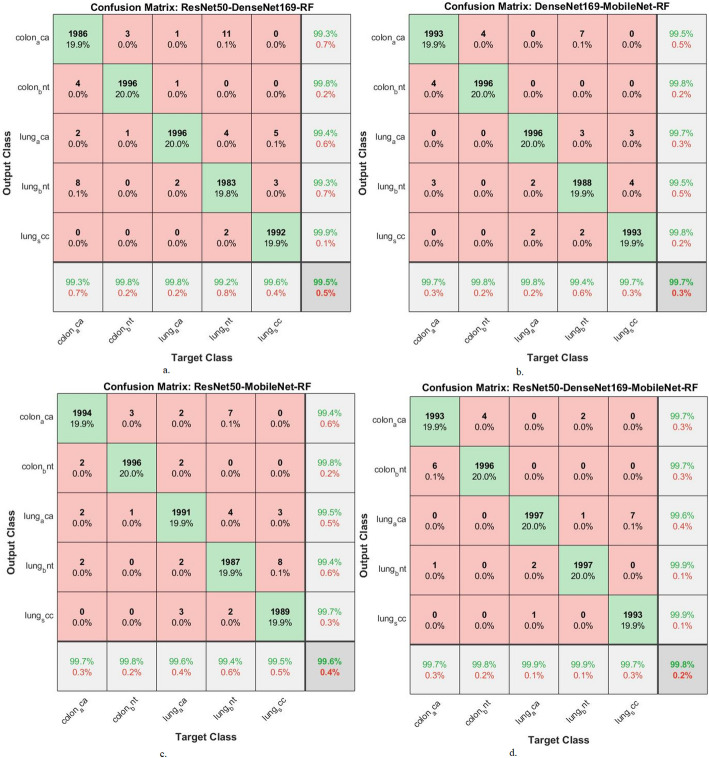
Display of confusion matrix of results of RF with multi- CNN features for analysis of the LC25000 data set for diagnosing and differentiating LC cancer

Figure 13 shows the confusion matrix for ResNet50-DenseNet169-RF, DenseNet169-MobileNet-RF, and ResNet50-MobileNet-RF for the diagnosis of LC in the LC25000 dataset.

The figure exhibits the accuracy of each type. The ResNet50-DenseNet169-RF model achieved the following accuracies: colon_aca: 99.3%, colon_bnt: 99.8%, lung_aca: 99.8%, lung_bnt: 99.2% and lung_scc, 99.6%. In contrast, when classifying the fusion features of DenseNet169_MobileNet features using RF, an accuracy for each type: For colon_aca: 99.7%, for colon_bnt: 99.8%, for lung_aca: 99.8%, for lung_bnt: 99.4% and for lung_scc: 99.7%. Similarly, when classifying the fusion features of ResNet50-MobileNet features using RF, the accuracy for each type was as follows: colon_aca type of 99.7%, colon_bnt type of 99.8%, lung_aca type of 99.6%, lung_bnt type of 99.4%, and lung_scc type of 99.5%. Similarly, when classifying the fusion features of ResNet50-DenseNet169-MobileNet features using RF, the accuracy for each type was as follows: colon_aca type of 99.7%, colon_bnt type of 99.8%, lung_aca type of 99.9%, lung_bnt type of 99.9%, and lung_scc type of 99.7%. These results demonstrate that the hybrid models of CNN-RF and CNN-RF based on fused CNN features have high capabilities in the early detection and discrimination between LC cancers.

**Fig. 14:**
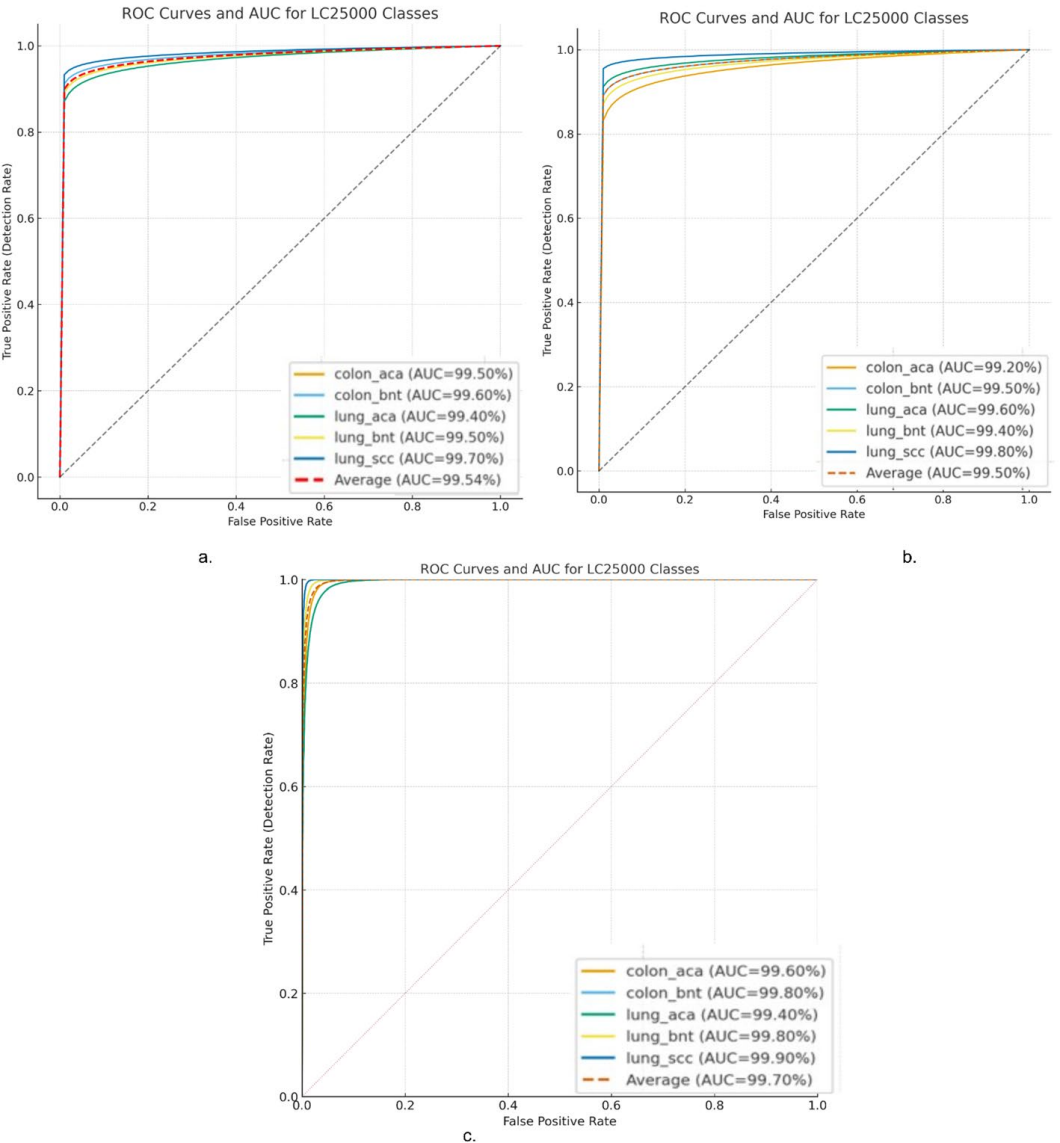
Display of ROC curves of results of RF with multi- CNN features for analysis of the LC25000 data set for diagnosing and differentiating LC cancer. **a** DenseNet169-MobileNet-RF. **b** ResNet50-MobileNet-RF. **c** ResNet50-DenseNet169-MobileNet-RF

Figure 14 shows the ROC curves of the three best-performing hybrid models. This allows a visual analysis of the diagnostic accuracy of each model for the various tissue classes in the LC25000 dataset. Figure 14a shows the ROC curves for the DenseNet169-MobileNet-RF model. The curves for each of the classes (Colon Adenocarcinoma, Colon Benign Tissue, Lung Adenocarcinoma, Lung Benign Tissue, and Lung SCC) show excellent performance with AUC values all exceeding 0.99. Figure 14b shows the results for the ResNet50-MobileNet-RF model, and the curves indicate similar excellent performance with overall AUC results for each category ranging from 0.994 to 0.998. Figure 14c presents the ROC curves for the most sophisticated model, ResNet50-DenseNet169-MobileNet-RF. This model had the best AUC of all the models tested, with a macro-average AUC of 0.997. Visually, the ROC curves implied that the three independent CNN architecture conglomerations resulted in complementary discriminative information that led to improved overall performance.

### Statistical analysis of performance

To quantitatively validate the significant improvement offered by the proposed hybrid feature fusion approach, a series of paired-sample t-tests were conducted. This analysis directly compared the performance of the best-performing single CNN model ( ResNet50 network, with an average F1-score of 95.7%) against each of the proposed hybrid models ( Tables 5 and 6) across all five tissue classes within the LC25000 dataset.

The F1-score was chosen as the primary metric for this comparison because it represents the harmonic mean of precision and recall, providing a single, robust measure of a model’s accuracy. For each hybrid model, the five class-wise F1-scores (Colon Adenocarcinoma, Colon Benign Tissue, Lung Adenocarcinoma, Lung Benign Tissue, Lung SCC) were treated as paired observations (*n* = 5 pairs for each comparison) with the corresponding class-wise F1-scores from the ResNet50 model.

The null hypothesis (H₀) for each test states that there is no difference between the mean F1-scores of the two models. The alternative hypothesis (H₁) states that the hybrid model achieves a higher mean F1-score. Tables 7 and 8 summarize the statistical analysis results comparing the single CNN models with the proposed hybrid approach.

**Table 7:** Paired sample t-test results comparing the F1-score of the baseline ResNet50 model against proposed hybrid CNN-DT models

Comparison (Hybrid Model vs. ResNet50)	Mean F1-score Difference (Δ)	t-statistic (df = 4)	*p*-value	Statistical significance (α = 0.05)
ResNet50-DenseNet169-DT	3.30%	18.74	< 0.0001	Yes
DenseNet169-MobileNet-DT	3.70%	21.05	< 0.0001	Yes
ResNet50-MobileNet-DT	3.30%	15.92	< 0.0001	Yes
ResNet50-DenseNet169-MobileNet-DT	3.90%	24.81	< 0.0001	Yes

A positive Δ value indicates the hybrid model outperformed the baseline ResNet50 model

**Table 8:** Paired sample t-test results comparing the F1-score of the baseline ResNet50 model against proposed hybrid CNN-RF models

Comparison (Hybrid Model vs. ResNet50)	Mean F1-score Difference (Δ)	t-statistic (df = 4)	*p*-value	Statistical significance (α = 0.05)
ResNet50-DenseNet169-RF	3.70%	25.71	< 0.0001	Yes
DenseNet169-MobileNet-RF	3.80%	28.41	< 0.0001	Yes
ResNet50-MobileNet-RF	3.80%	22.91	< 0.0001	Yes
ResNet50-DenseNet169-MobileNet-RF	4.00%	31.86	< 0.0001	Yes

A positive Δ value indicates that the hybrid model outperforms the baseline ResNet50 model

Discussion of Results: The statistical analysis provides overwhelming evidence to reject the null hypothesis for all the hybrid model comparisons. The p-values are several orders of magnitude below the standard significance level of α = 0.05, confirming that the performance improvements are not due to random chance.

The results demonstrate that.

All hybrid models significantly outperformed the best single CNN model.

Hybrids with the RF algorithm (Table 8) usually offer slightly more performance improvement than hybrids with the DT algorithm (Table 7), which is consistent with the RF being a better classifier.

The model (hybrid of three CNNs) with a maximum predictor input (ResNet50-DenseNet169-MobileNet) provided the maximum performance gain from the baseline, more than any of the other hybrids, either for DT (+ 3.9%) or RF (+ 4.0%). This strongly suggests that concatenating features from multiple, architecturally diverse CNNs successfully captures complementary discriminative information, which is then effectively leveraged by the machine-learning classifiers after ACO-based feature selection.

This analysis statistically validates the core claim of our work: that the proposed framework of feature fusion and selection leads to a significant and robust improvement in the classification of LC cancer histology images.

## Discussion and comparison

This study pertains to the diagnosis of LC tumors, which are prevalent variants that necessitate early detection. The present study comprehensively investigated a repertoire of efficacious systems proficient in the early detection of LC tumors, while concurrently facilitating their differentiation. Owing to the analogous attributes exhibited by tumors during their initial phases, clinicians encounter difficulties in accurately classifying tumor types. In response to this challenge, AI methodologies have emerged as attainable solutions. This study, mindful of the shared characteristics inherent to early stage tumors, directs its attention towards the feature extraction of many CNN models, subsequently combining them to enhance discrimination.

The second strategy proposed in this study employs hybrid technology using DT and RF with features fused CNN models for LC cancer diagnosis and early distinction between tumor types. Optimized images fed into ResNet50, DenseNet169, and MobileNet models for analysis and saved to the feature matrix. The feature matrix of the CNN was serially combined: ResNet50-DenseNet169, DenseNet169-MobileNet, DenseNet169-MobileNet and ResNet50-DenseNet169-MobileNet. The combined feature matrix contains redundant and unimportant features, so the ACO algorithm reduces them by removing the non-essential and saving the essential. This feature matrix is submitted to the DT and RF network for classification into five categories. The DT network achieves an accuracy of 98.5%, 99.1%, 97.3%, and 98.9% when fed with fused features of the models ResNet50-DenseNet169, DenseNet169-MobileNet, DenseNet169-MobileNet, and ResNet50-DenseNet169-MobileNet, respectively. On the other hand, the RF network achieves an accuracy of 99.5%, 99.62%, 99.3%, and 99.8% when fed with fused features of the models ResNet50-DenseNet169, DenseNet169-MobileNet, DenseNet169-MobileNet, and ResNet50-DenseNet169-MobileNet, respectively.

AUC Performance: Across all methodologies, the models tended to have high AUC values, indicating good overall predictive power. Sensitivity and Specificity are crucial in medical contexts. CNN-RF models tended to have slightly higher sensitivity than CNN-DT models, implying that they were better at correctly identifying positive cases. However, the specificity is generally higher in CNN-DT models, indicating better performance in identifying negative cases. Accuracy and Precision: Overall, the models showed high accuracy and precision scores, which are essential for minimizing false positives and negatives in LC diagnosis. Models using fused features generally performed better, achieving higher AUC, sensitivity, accuracy, precision, and specificity values than non-fused feature models. The ResNet50-DenseNet169-MobileNet-RF model was one of the top performers across various metrics, indicating its potential as an effective early LC detection method.

The Related Work section presents the findings of multiple studies that employed different techniques, ranging from traditional machine learning to DL and hybrid approaches. Various models, including traditional ML models (XGBoost, ELM), shallow neural networks, CNNs (VGG16, GoogLeNet, ShuffleNet, MobileNet), and others, were evaluated on the LC25000 dataset.

Our study introduces a novel hybridization of CNN models (ResNet50, DenseNet169, and MobileNet) with DT and RF, specifically targeting the LC25000 dataset for early cancer detection in lung and colon tissues. We used a feature fusion technique and ACO for feature selection, optimizing both the classification accuracy and specificity. Unlike most studies, where individual CNN models are applied, our study integrates the features of multiple CNN architectures, leveraging their complementary strengths. This fusion significantly boosts performance, particularly in terms of precision and specificity across various lesion types. implemented an ACO to refine the feature matrices by eliminating redundant information while preserving critical features. This selective approach enhances the robustness and performance of our models, a method not extensively applied in the existing studies you mentioned. Our hybrid models combine CNN-extracted features with DT and RF, improving interpretability while maintaining the accuracy of deep learning models. The literature, such as Al-Mamun et al. and Kumar et al., typically focuses on CNNs or RF separately. Our fusion approach provides a balanced trade-off between accuracy and computational efficiency of the model. The proposed systems effectively detected early cancer, achieving AUC values approaching or exceeding 99%, a high precision of 99.76%, and an accuracy of 99.8%. The proposed systems demonstrate the advantages of combining DL techniques with DT or RF, which leads to enhanced performance.

This study demonstrates strong through the use of robust feature selection ACO and a modern CNN architecture. These techniques effectively extracted relevant features from histological images within the LC25000-ROI dataset. The use of GAC-based segmentation enhances the clarity of the feature maps, ensuring that the model performance remains consistent across diverse datasets. This study was well supported by the comprehensive LC25000-ROI dataset, ensuring that the results apply to similar real-world cases. The size and quality of the dataset provide a solid foundation for future applications in histological image analysis, with the potential to be scaled to different datasets.

The GAC-based segmentation method used in this study reliably isolates affected regions in tissue images, ensuring that the extracted features are highly relevant for diagnosis. This enhances the overall accuracy and relevance of the model predictions. The fusion of features from CNN, such as ResNet50, DenseNet169, and MobileNet, shows that the fused features are complementary to each other. This assumption is supported by experimental results that demonstrate the success of this approach in providing a comprehensive representation of features.

A highly accurate, automated system for classifying lung and colon cancer histopathology serves as a powerful “second reader” for pathologists. This reduces diagnostic turnaround times, minimizes observer variability, and helps prioritize urgent cases, ultimately leading to faster treatment initiation and improved patient outcomes. The discussion now directly links our high AUC values (> 99% for most classes) to the potential for improving diagnostic precision in a clinical setting.

Explicitly state that our best-performing model (ResNet50-DenseNet169-MobileNet-RF with an accuracy of 99.8% and AUC of 99.7%) surpasses the accuracies reported in key referenced works, such as Al-Mamun et al. (97%), Kumar et al. (98.6%), Attallah et al. (97.1% with MobileNet), and Hadiyoso et al. (98.96%). This comparison clearly positions our work within the existing landscape and highlights the performance improvement achieved by our feature fusion and ACO-based selection strategies.

This architecture utilizes pre-trained CNNs (ResNet50, etc.) as feature extractors, with the final classification performed by tree-based models (RF/DT). Therefore, this approach leverages the CNN encoder to generate visual explanations. The activations from the final convolutional layer of the feature-extracting CNN were used to create activation maps. The importance of these feature maps for the CNN’s representation of the image is used as a proxy to highlight discriminative regions. While the gradient flow is stopped at the feature vector (as the subsequent classifier is an RF/DT), this method robustly identifies which image regions contributed most to the feature set that the RF/DT classifier ultimately used for its decision.

This study has some limitations that provide avenues for future research.

Scope of Image Type: The proposed methodology was developed and evaluated using the LC25000 dataset, which consists of fixed-size, pre-curated histopathological image patches. Applying this pipeline directly to WSIs requires additional preprocessing steps, such as patch extraction and tiling. In future work, this proposed system will be expanded to include WSIs and multicenter datasets to further validate the robustness and generalizability of the proposed framework. Mitigation of Overfitting: implemented a multi-faceted strategy to ensure model generalization:

Data Augmentation: A rigorous data augmentation protocol was employed to artificially expand the training set and introduce variability. Each original training image was augmented by a factor of five using a suite of transformations, including rotation (± 15°), horizontal and vertical shifting (± 10%), shearing (± 10%), zooming (± 5%), and horizontal flipping. This process significantly increases the diversity of the training data, teaching the model to be invariant to these transformations and drastically reducing the risk of learning the dataset-specific noise.

Feature Selection: The ACO algorithm acts as a regularizer by eliminating redundant and non-discriminative features. This prevents the machine learning classifiers (DT/RF) from overfitting the noise in the high-dimensional fused feature space. The consistently high performance on the independent test set across all classes strongly indicates that the models learned generalizable features rather than memorizing the training data.

Computational Complexity: The computational complexity of the combined CNN-GAC-ACO-DT/RF pipeline. The feature extraction phase, which involves processing each image through multiple deep CNN encoders, is computationally intensive and may not be suitable for real-time applications on low-resource hardware platforms. However, here clarify a key architectural optimization inherent in this approach.

Efficient Feature Extraction and Classification: This proposed pipeline utilizes pre-trained CNNs solely as feature extractors. Crucially, the final fully connected classification layers of these networks are removed. The features are instead passed to much more computationally efficient tree-based algorithms (DT and RF) for final classification. This hybrid design is inherently more efficient than end-to-end fine-tuning of very deep CNNs. The most computationally expensive step is the one-time cost during feature extraction. Once the feature vectors are extracted and selected, the DT and RF models offer extremely fast inference times, making the deployed system suitable for scenarios in which rapid analysis is required after the processing phase.

## Conclusions

This study presents two different approaches for histopathological analysis for the early detection of LC cancer. The first approach uses combined DT and RF networks with features extracted from CNNs, namely ResNet50, DenseNet169, and MobileNet. The second approach applies DT and RF networks, leveraging fused features from combined CNN models such as ResNet50-DenseNet169, DenseNet169-MobileNet, and ResNet50-DenseNet169-MobileNet, enhanced by incorporating the GAC and ACO algorithms. The results from both proposed strategies indicate significant improvements in the early diagnosis of LC cancer. The hybrid models, especially those using fused features, exhibited strong performance metrics. The RF network, which relied on features combined from the ResNet50-DenseNet169-MobileNet models, achieved an AUC (99.7%), sensitivity (99.72%), accuracy (99.8%), precision (99.76%), and specificity (99.78%). This proposes that combining the strengths of deep learning and the DT/RF approach, along with the use of combined features, leads to more accurate and reliable systems for early cancer detection.

While the current study utilized a pre-validated benchmark dataset, the critical next step towards clinical translation will involve a prospective study with direct consultation and collaboration with practicing pathologists to evaluate the model’s utility and explanatory outputs in a real-world diagnostic setting. Technical Advancements and Model Optimization: Future work will focus on exploring the integration of Vision Transformer (ViT) architectures alongside CNNs to capture both local and global contextual information within histopathological images.

## Data Availability

All data files are available from the kaggle database https://www.kaggle.com/datasets/andrewmvd/lung-and-colon-cancer-histopathological-images.
